# Perturbations in Histidine Biosynthesis Uncover Robustness in the Metabolic Network of *Salmonella enterica*

**DOI:** 10.1371/journal.pone.0048207

**Published:** 2012-10-25

**Authors:** Mark J. Koenigsknecht, Jennifer A. Lambrecht, Luke A. Fenlon, Diana M. Downs

**Affiliations:** Department of Bacteriology, University of Wisconsin-Madison, Madison, Wisconsin, United States of America; University of Florida, United States of America

## Abstract

Phosphoribosylamine (PRA) is an intermediate in the biosynthetic pathway that is common to thiamine and purines. Glutamine phosphoribosyl pyrophosphate (PRPP) amidotransferase is the product of the *purF* gene in *Salmonella enterica* and catalyzes the synthesis of PRA from PRPP and glutamine. Strains lacking PurF require exogenous addition of purines for growth. However, under some growth conditions or with specific secondary mutations these strains grow in the absence of exogenous thiamine. Mutant alleles of *hisA*, which encodes 1-(5-phosphoribosyl)-5-[(5-phosphoribosylamino) methylideneamino] imidazole-4-carboxamide (ProFAR) isomerase, allowed PurF-independent PRA formation. The alleles of *hisA* that suppressed the requirement for exogenous thiamine resulted in proteins with reduced enzymatic activity. Data presented here showed that decreased activity of HisA altered metabolite pools and allowed PRA formation from ProFAR. Possible mechanisms of this conversion were proposed. The results herein emphasize the plasticity of the metabolic network and specifically highlight the potential for chemical syntheses to contribute to network robustness.

## Introduction

Discrete biochemical pathways define the framework of metabolism. Superimposed on this framework is a network of interactions mediated by metabolites. Our understanding of the metabolic framework has been achieved through decades of biochemical and genetic studies, many of them in bacterial systems. In contrast, efforts to identify the network of interactions mediated by metabolites and define the significance of these interactions to the fitness of the organism are in the early stages. Thiamine biosynthesis in *Salmonella enterica* has proven to be a productive model system to study metabolic integration and robustness (reviewed in [1]). Basic research in microbiology has a long history of using mutational analysis *in vivo* to gain insights into the function of the wild-type system [2]. We have shown that thiamine biosynthesis in *S. enterica* is amenable to *in vivo* analyses, making it a powerful system to query an organism about the characteristics of the naturally occurring metabolic network and dissect its potential.

**Figure 1:**
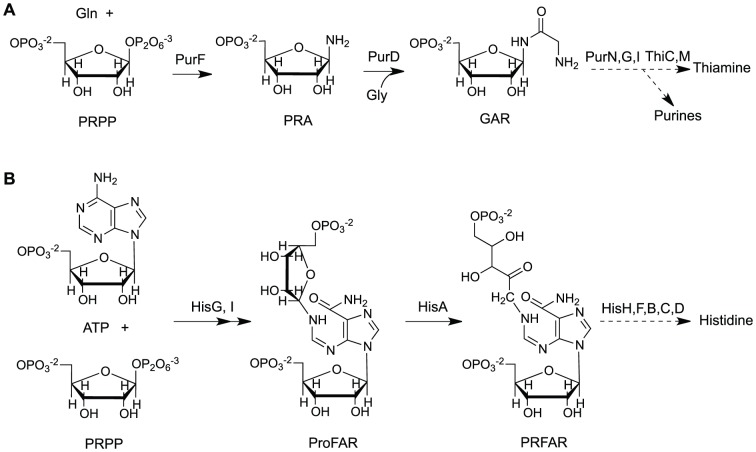
The biosynthetic pathways for thiamine and histidine in *S. enterica*. Panel A shows relevant steps from the thiamine biosynthetic pathway while panel B shows relevant steps in the histidine synthetic pathway, both in *S. enterica*. The enzymatic steps that lead to the formation of their respective end products are show. Abbreviations: Gln, glutamine; PRPP, phosphoribosyl pyrophosphate; PRA, phosphoribosylamine; Gly, glycine; GAR, glycineamide ribonucleotide; ProFAR, 1-(5-phosphoribosyl)-5-[(5-phosphoribosylamino)methylideneamino]imidazole-4-carboxamide; PRFAR, 5-[(5-Phospho-1-deoxyribulos-1-ylamino)methylideneamino]-1-(5-phosphoribosyl)imidazole-4-carboxamide.

Thiamine is an essential cofactor synthesized *de novo* by bacteria, archaea, yeast and plants. In bacteria, the pyrimidine moiety of thiamine is synthesized from a branch point metabolite of the purine biosynthetic pathway. The product of the *purF* gene, glutamine- phosphoribosyl pyrophosphate (PRPP) amidotransferase (EC 2.4.2.14), catalyzes the first step in the shared purine/thiamine pathway and synthesizes phosphoribosylamine (PRA) from PRPP and glutamine (Figure 1A). As expected, strains lacking PurF require exogenous purines. However, under certain growth conditions (or with specific secondary mutations) *purF* mutant strains can generate sufficient thiamine to allow growth without exogenous addition of this vitamin [1]. Such growth reflects the robustness of the metabolic network surrounding PRA and indicates the existence of PurF-independent mechanisms to generate this metabolite. Thus far, no PurF-independent mechanism has generated sufficient PRA to satisfy the cellular purine requirement.

PRA can be synthesized in the absence of PurF by altering metabolic flux in distinct pathways and thus increasing the pool size of relevant metabolic intermediates. Genetic and biochemical studies showed that accumulation of phosphoribosyl anthranilate (PR-anthranilate), an intermediate in tryptophan biosynthesis, allowed formation of thiamine in the absence of PurF [3]. In this case, the unstable PR-anthranilate decomposed into ribose-5′-phosphate (R5P) and anthranilate, and the newly available R5P reacted non-enzymatically with ammonia in the medium to form PRA [3]. In a separate study, mutations that compromised the essential enzyme PRPP synthase (PrsA) supported non-enzymatic PRA formation [4]. PrsA combines R5P and ATP to generate PRPP. Therefore, decreased activity of the PrsA enzyme resulted in accumulation of R5P that was then available for non-enzymatic formation of PRA when sufficient ammonia was present [4]. In each of these, as well as other cases, the formation of PRA depended on the accumulation of available R5P non-enzymatically reacting with ammonia in the medium. Together these studies illustrated the potential for the perturbation of steady state pathway flux to alter metabolite availability, which could result in non-enzymatic synthesis of PRA. Other examples of non-enzymatic metabolite synthesis have been described [5].

In strains lacking the *ridA* (previously *yjgF*) gene, a distinct mechanism of PRA synthesis that involved the tryptophan biosynthetic enzyme anthranilate phosphoribosyltransferase (TrpD; EC 2.4.2.18) occurred that was independent of ammonia in the medium [1], [6]. In this case, the dehydration of threonine by IlvA (EC 4.3.1.19) produced an enamine intermediate that was combined with PRPP by TrpD to form an unstable product. This metabolite was then broken down directly into PRA [7], [8]. In strains with wild-type RidA, the threonine-derived enamine metabolite was not available for TrpD, and PRA formation did not occur by this mechanism. To date, this is the only example of PRA synthesis independent of PurF that depends on an enzyme utilizing a non-native substrate to produce PRA without requiring R5P and ammonia intermediates.

The study described here was initiated to extend our understanding of PurF-independent PRA formation and specifically to address if additional pradigms of robustness exist in this node of the network. Genetic analyses described herein identified the histidine biosynthetic intermediate 1-(5-phosphoribosyl)-5-[(5-phosphoribosylamino)methylideneamino]imidazole-4-carboxamide (ProFAR) as a precursor in the formation of PRA. We suggest that ProFAR-dependent synthesis of PRA is due to enzymatic breakdown of ProFAR either directly to PRA or to R5P and ammonia which would then non-enzymatically combine to form PRA.

## Materials and Methods

### Bacterial strains, media, and chemicals

Unless otherwise indicated, the strains used in this study are derivatives of *S. enterica* serovar Typhimurium strain LT2 and their genotypes are listed in Table 1. MudJ refers to Mud1734 [9] and Tn*10d*(Tc) refers to the transposition-defective mini-Tn*10*(Tn*10*Δ*16*Δ*17*) [10] which have been described elsewhere. Culture media supplies were obtained from Difco (Sparks, MD). Buffers and salts were obtained from Fisher Scientific (Pittsburgh, PA), and all other chemicals were obtained from Sigma-Aldrich (St, Louis, MO). All restriction enzymes used were from Promega (Madison, WI). DNase I (EC 3.1.21.1), lysozyme (EC 3.2.1.17), and inorganic pyrophosphatase (EC 3.6.1.1) were obtained from Sigma-Aldrich. No-carbon E medium (NCE) of Vogel and Bonner [11], [12] supplemented with 1 mM MgSO_4_, trace minerals, and 11 mM glucose was used as minimal medium. Trace minerals were dissolved in 1.5 mM nitrilotriacetic acid pH 7 as 1000x stock adapted from Balch *et al* [13]. Nitrogen and carbon-free salts medium (N^−^C^−^) [14] supplemented with trace minerals, 11 mM glucose, and 1 mM glutamine was used as limiting nitrogen minimal medium. Difco nutrient broth (8 g/liter) with NaCl (5 g/liter) was used as rich (NB) medium. Luria-Bertani broth was used for experiments involving molecular biology and plasmid isolation. Difco BiTek agar was added (15 g/liter) for solid medium. Superbroth (32 g tryptone, 20 g yeast extract, 5 g NaCl, and 1 ml of 5 N NaOH/liter) was used as rich (SB) medium for protein purification. When present in the culture medium and unless otherwise stated, the compounds were used at the following final concentrations: adenine, 0.4 mM; thiamine, 100 nM; histidine, 0.1 mM; tryptophan, 0.1 mM; isopropyl- β-d-thiogalactopyranoside (IPTG), 0.5 mM. The final concentrations of the antibiotics in rich media were as follows: tetracycline (Tc), 20 µg/ml; kanamycin, 50 µg/ml; ampicillin (Amp), 150 µg/ml; and chloramphenicol (Cm), 20 µg/ml.

**Table 1:** Bacterial strains.

Strain	Genotype
DM728	*purF2085 gnd174*::MudJ^a^
DM1936	*purF2085*
DM9972	*purF2085 gnd181 trp-3618 hisA1455* b
DM10000	Wild type
DM10350	*purF2085 gnd174*::MudJ *hisA1451*
DM10351	*purF2085 gnd174*::MudJ
DM10352	*purF2085 gnd174*::MudJ *hisA1452*
DM10374	*purF2085 trp-3618 gnd174*::MudJ
DM10406	*purF2085 gnd174*::MudJ *hisA1462*
DM10425	*purF2085 gnd174*::MudJ *hisA1467*
DM10427	*purF2085 gnd174*::MudJ *hisA1468*
DM10429	*purF2085 gnd174*::MudJ *hisA1459*
DM10922	*purF2085 gnd174*::MudJ *hisG1102 hisA3000*
DM10923	*purF2085 gnd174*::MudJ *hisA3000*
DM10924	*purF2085 gnd174*::MudJ *hisG1102*
DM10928	*purF2085 gnd174*::MudJ *hisG1102 hisI99*
DM10931	*purF2085 gnd181 zxx-4102*::Tn*10*d(Tc)^c^ *hisG1102 his-2652*
DM10994	*purF2085 gnd181 zxx-4102*::Tn*10*d(Tc) *hisG1102 his-2652* pSU-*hisI*
DM10969	*purF2085 gnd174*::MudJ *hisA10501*
DM10973	*purF2085 gnd174*::MudJ *hisA10505*
DM10975	*purF2085 gnd174*::MudJ *hisA10506*
DM11012	*purF2085 gnd174*::MudJ *hisA10508*
DM11014	*purF2085 gnd174*::MudJ *hisA10509*
DM11069	*purF2085 gnd174*::MudJ *hisA1460*
DM11071	*purF2085 gnd174*::MudJ *hisA10521*
DM11075	*purF2085 gnd174*::MudJ *hisA10523*
DM11077	*purF2085 gnd174*::MudJ *hisA10524*
DM11081	*purF2085 gnd174*::MudJ *hisA10526*
DM11136	*purF2085 gnd174*::MudJ *hisG1102 hisF109*
DM11185	*purF2085 gnd174*::MudJ *hisA1451 hisG10527*
DM11234	*purF2085 gnd174*::MudJ *hisO1242*
DM11525	*purF2085 gnd174*::MudJ *hisA1451 trp-3618*::Cm
DM11806	*purF2085 gnd181 hisA1451 proY2301*::Tn*10*d(Tc)
DM12277	*purF2085 gnd181 hisA1451 yciB65*
JW2001	*E. coli* AG1 (Stratagene) pCA24N-*hisG* (-gfp) [25]
JW2008	*E. coli* AG1 (Stratagene) pCA24N-*hisI* (-gfp) [25]

a MudJ refers to the Mud1734 transposon [9].

b Allele numbers for *hisA* in the 1400 s were issued by the Salmonella Genetic Stock Center as *hsi* alleles for historical reasons. For simplicity, we have used the *his* designation herein.

c Tn*10d*(Tc) refers to the transposition-defective mini-Tn*10*(Tn*10Δ16Δ17*) [10].

### Growth quantitation

Cells from overnight cultures in NB medium were pelleted and resuspended in an equal volume of saline (85 mM NaCl). An aliquot of 100 µl of this suspension was used to inoculate 5 ml of the appropriate minimal medium. Cell density (OD_650_) was monitored every hour in a Thermo Electron Corporation Spectronic 20D+ apparatus while incubating at 37°C with shaking at 200 rpm. Alternatively, 5 µl of the cell suspension was used to inoculate 195 µl of the appropriate minimal medium contained in each well of a 96-well microtiter plate. Growth at 37°C was monitored using a microplate spectrophotometer Spectra-Max Plus. For each case, the specific growth rate was determined as *μ* = ln(*X/X_o_*)/*T*, where *X* is the *A_650_* value during the linear portion of growth and *T* is time in hours.

### Genetic techniques

Transductional crosses were performed using the high-frequency general transducing mutant of bacteriophage P22 (HT105/1, *int*-201) [15], [16]. Transductants were purified by single colony isolation on non-selective green indicator plates [17]. Identification of phage-free transductants was done by cross-streaking against bacteriophage P22. Strains with multiple mutations were constructed using standard genetic techniques. The creation of strains with the *hisG1102* allele was confirmed by sequence analysis of *hisG*. The *hisG1102* allele has a C-to-A base substitution at nucleotide 791 that resulted in an A264E variant protein. Additionally, sequence analysis was used to confirm the presence of *hisO1242* [18].

### Mutant isolation

Strain DM10374 (*purF2085 trp-3618 gnd174*::MudJ) was used for isolation of suppressor mutations that allowed PurF-indpendent thiamine synthesis. Deletion of the tryptophan operon prevented recovery of *trpC* mutations, that have been previously characterized to allow for PurF-independent PRA formation [3] and the insertion in *gnd* increased the stringency of the selection by eliminating background non-enzymatic PRA synthesis [19]. Neither mutation was required for the PurF-independent PRA synthesis resulting from the *hisA* alleles isolated herein. An aliquot (100 µl) of a saline suspension of DM10374 was spread on NCE medium supplemented with adenine and tryptophan. After 72 hours of incubation at 37°C, mutations allowing growth without exogenous thiamine arose at a frequency of ∼5×10^−7^. Linkage between the histidine operon and *gnd174*::MudJ was exploited to move the causative mutation into strain DM1936 (*purF2085*) background.

Strain DM11525 (*purF2085 gnd174*::MudJ *hisA1451 trp-3618*::Cm) was used to isolate spontaneous suppressor mutations that allowed growth in the presence of 100 µM histidine. The most commonly isolated mutants contained feedback resistant alleles of *hisG*, as determined by excretion of histidine into the media by these strains to [20]. One such mutant, *hisG10527*, was sequenced and had a C736A substitution that resulted in a P246T variant protein. Subsequent screens included 600 µM of the false feedback inhibitor, thiazolealanine, to screen against these mutants [21]. A pool of random Tn*10* insertions were used to map the causative mutation in two of the resulting isolates. Both revertants were subsequently identified as frameshift mutations of *yciB* (STM1735). An insertional deletion of *yciB* was constructed (*yciB65*) by the method of Datsenko [22]. In the process of mapping the lesions in *yciB*, a Tn*10* insertion in *proY* (*proY2301*::Tn*10d*(Tc)) was identified that eliminated the effect of histidine on thiamine synthesis.

### Molecular biology

Amplification of *hisA* and *hisI* from *S. enterica* LT2 chromosomal DNA was performed by PCR using Herculase II Fusion DNA Polymerase (Agilent, Santa Clara, CA). The primers used for amplification of *hisI* were HisI5′ (5′ GCAAATCATCAATATTGGCG 3′) and HisI3′ (5′ TGGTCGGCTACGACAGGTA 3′). PCR conditions were as follows: denaturation at 95°C for 30 seconds, annealing at 50°C for 30 seconds, and extension at 72°C for 30 seconds. The primers used for amplification of *hisA* were HisA5′NdeI (5′ CATATGATTATTCCGGCATTAGA 3′) and HisA3′BamHI (5′ GGATCCTTATACGTTTTGCCAGC 3′). PCR conditions were as follows: denaturation at 95°C for 30 seconds, annealing at 58°C for 30 seconds, and extension at 72°C for 30 seconds.

The resulting 738 bp fragment for *hisI* and the 745 bp fragments for *hisA* were PCR purified using a Qiagen (Germantown, MD) PCR Purification Kit. The PCR products for *hisA* and *hisI* were blunt-end ligated into pSU18 cut with SmaI [23] using T4 DNA ligase (Promega). *E. coli* DH5α was electroporated with the ligation mix and electroporants were tested for the presence of the gene by a restriction enzyme digestion of the resulting plasmid. In each case sequence analysis confirmed the correct placement of the gene with respect to the Lac promoter and that the respective gene was wild-type.

### Protein purification

All proteins were purified with a similar protocol except for glycineamide ribonucleotide (GAR) synthetase (EC 6.3.4.13), PurD, which was purified as previously described [24]. Strains containing pCA24N-*hisG* (JW2001) and pCA24N-*hisI* (JW2008) [25] were used for HisG and HisI (bifunctional phosphoribosyl-ATP pyrophosphohydrolase and phosphoribosyl-AMP cyclohydrolase (EC 3.6.1.31, 3.5.4.19)) purifications, respectively. Each strain was grown in 3 liters SB+Cm at 37°C with shaking. When the cultures reached an absorbance (OD_650_) of ∼0.5, IPTG was added to a final concentration of 0.5 mM. After 14 hours of incubation, the cells were pelleted by centrifugation and the cell pellet (∼8 g) was stored at −80°C.

All subsequent steps were performed at 4°C. The binding buffer used was the following: 50 mM potassium phosphate pH 7.5, 100 mM KCl, and 5% glycerol. Wash buffer contained binding buffer with 10 mM imidazole and 500 mM KCl. Elution buffer contained wash buffer with 500 mM imidazole. The frozen cell pellet was weighed and washed in an equal volume (wt/vol) of binding buffer, centrifuged at 42,500×*g* for 10 min, then resuspended in an equal volume of binding buffer. DNase I and lysozyme were each added to a final concentration of 0.01 mg/ml and the suspension was incubated on ice for 10 min. The cell solution was passed through a French pressure cell at 18,500 psi three times. The extract was centrifuged at 42,500×*g* for 45 min and the supernatant was passed through a 0.45 µm filter. The sample was loaded on a column packed with Qiagen Ni-NTA Superflow resin (4.5 ml) that had been equilibrated with at least 10 column volumes (CV) of binding buffer. After the sample was loaded, wash buffer was run over the column until there was no detectable absorbance at 280 nm. A 10 CV gradient from 0–100% elution buffer was applied and both proteins were eluted from the column by 400 mM imidazole. Fractions containing the protein of interest were concentrated at 30 psi under Argon gas using a 10,000 MWCO membrane, Amicon YM10, from Millipore (Billerica, MA). The proteins were dialyzed overnight in binding buffer, frozen in liquid nitrogen, and stored at −80°C.

### ProFAR synthesis and purification

ProFAR was synthesized and purified using a modification of a reported protocol [26]. Briefly, 50 mM potassium phosphate pH 7.5, 10 mM MgCl_2_, 1 mM EDTA, 5 mM ATP, 6.6 mM PRPP, 10 U inorganic pyrophosphatase, 2.5 µg HisG, 4.0 µg HisI were incubated for 12 hours at 28°C, in a final volume of 8 ml. Proteins were removed with a 10,000 MWCO filter and the flow-through was diluted 1∶5 into 50 mM NaHCO_3_ pH 7.5 and applied to a 2.0 ml Q-Sepharose column equilibrated with 50 mM NaHCO_3_. A gradient from 50 to 175 mM NaHCO_3_ was applied over 10 CV. ProFAR eluted at ∼150 mM NaHCO_3_, and was followed by absorbance at 280 nm. Fractions containing ProFAR were lyophilized and the powder was resuspended in 100 mM triethylammonium acetate (TEAA) buffer pH 7.5 and 1 M NaOH at a 1∶1 ratio yielding a final pH of 7.5. This solution was loaded onto a C18 Gemini column (5 µm, 110 A, 150 mm by 4.6 mm) from Phenomenex (Torrance, CA) on an HPLC system operating at 1 ml/min with 100 mM TEAA/2% methanol as the eluent. In this system ProFAR eluted at 4.9 minutes as detected by absorbance at 290 nm. ProFAR used in the study was purified using this system and then lyophilized. The powder was resuspended in 50 mM potassium phosphate buffer pH 7.5, or alternatively, water pH 4. The concentration of ProFAR was determined using the reported molar extinction coefficient of 8000 M^−1^ cm^−1^ at 290 nm [27]. The identify of ProFAR was confirmed by UV spectrum, co-injection with an authentic standard in HPLC, and negative time-of-flight mass spectral analysis (expected, *m/z* 576.0822; observed, *m/z* 576.0817).

## Results

### Mutant alleles of *hisA* allow PurF-independent PRA formation in vivo

Strain DM10374 contains deletions of the *purF* gene and *trpEDCBA* operon and carries an insertion in the *gnd* locus. This strain is unable to grow on minimal medium with adenine and tryptophan due to a requirement for thiamine caused by the lack of PurF. Spontaneous mutations arose at a frequency of ∼5×10^−7^ that allowed growth after three days incubation at 37°C. The causative mutation in each revertant strain was linked to the *gnd* locus and subsequently mapped to the histidine operon. Sequence analysis determined that 27 out of 28 independent mutations that allowed PurF-independent PRA synthesis in this genetic background were in the *hisA* locus. Each of the 27 independent *hisA* mutants carried one of 17 mutant alleles that resulted in substitution of a conserved residue (>15/30 representative Gammaproteobacteria species) in the protein (Table 2). No substitutions in essential catalytic residues [28] were identified, consistent with the screen employing a growth medium that lacked histidine. The *hisA* gene encodes 1-(5-phosphoribosyl)-5-[(5-phosphoribosylamino)methylideneamino]imidazole-4-carboxamide (ProFAR) isomerase (HisA, EC 5.3.1.16), which catalyzes the third step in the histidine biosynthetic pathway (Figure 1B). The remaining suppressor mutation was in *hisI* (HisIL19H) affecting the PR-ATP hydrolase domain previously attributed to *hisE*. Unlike the *hisA* suppressors, this effect could not be recapitulated with a null allele of *hisE* or *hisI*. Based on these data we concluded the mechanism of suppression by this allele was different than that of the *hisA* alleles, and it was not pursued further in this study.

**Table 2:** Diverse mutations in *hisA* allow PurF-independent PRA synthesis.

*hisA* allele	DNA mutation^a^	Protein variant	# isolates^b^
*hisA10501*	636 to 638 del	Δ210	1
*hisA1451*	713 C to A	A238D	7
*hisA10509*	199 to 210 del	Δ67–70	1
*hisA1460*	44 G to A	R15H	2
*hisA10506*	491 G to A	G164D	1
*hisA1468*	305 G to A	G102D	1
*hisA1462*	293 G to C	R98P	1
*hisA10523*	736 T to C	Stop to Q	1
*hisA10505*	233 T to A	V78D	1
*hisA10521*	491 G to T	G164V	1
*hisA1459*	150 T to G	D50E	2
*hisA10508*	11 C to T	P4G	2
*hisA1452*	542 G to C	G181A	2
*hisA1467*	11 C to A	P4L	1
*hisA10524*	743 to 748 del	Δ215–216	1
*hisA1455*	658 to 666 del	Δ220–222	1

a From the annotated LT2 genome, NCBI GeneID: 1253299. Numbering starts at the first nucleotide of the coding sequence for HisA. Δ: Deletion

b Independent isolates.

### Mutations in *hisA* are recessive and result in a protein with reduced activity

**Figure 2:**
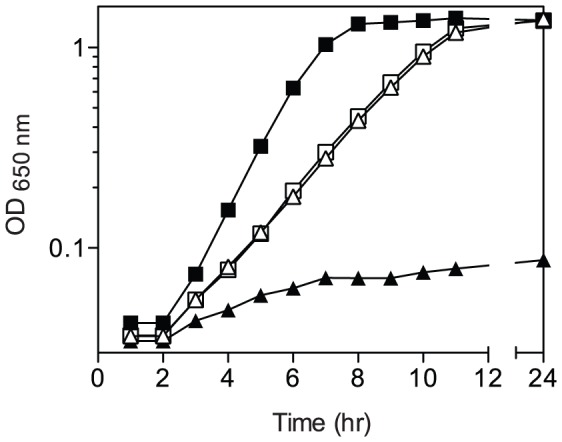
Growth analysis of *hisA* mutant strain. Growth curves were performed by monitoring optical density (OD) at 650 nm. Shown is a *purF2085 gnd174*::MudJ *hisA1451* (DM10350) strain grown in minimal glucose adenine medium (open triangle), with thiamine (open square), with histidine (filled triangle), and with histidine and thiamine (filled square).

The ability of a representative *hisA* mutant to allow PurF-independent PRA formation is shown as growth in Figure 2. Two results allowed the conclusion that the *hisA* alleles isolated were recessive and resulted in protein variants with decreased catalytic efficiency. First, when a wild-type copy of *hisA* was expressed *in trans*, strains had no detectable growth after 24 hours in the absence of exogenous thiamine. This result indicated the thiamine auxotrophy of the parental strain had been restored. Second, growth of the *hisA* mutant strains was stimulated by exogenous histidine in the presence of thiamine (Table 3). Together these results indicated the mutant alleles decreased, but did not eliminate flux through the histidine biosynthetic pathway.

**Table 3:** Growth rates of some *hisA* mutant strains are increased by exogenous histidine.

Strain	Genotype	No addition	+Thi	+Thi+His
DM10000	Wild-type	0.62±.03	0.65±.01	0.57±.01
DM728	*purF gnd*	NG^a^	0.56±.01	0.49±.01
DM11077	*purF gnd hisA10524*	0.43±.01	0.57±.01	0.48±.01
DM11081	*purF gnd hisA10526*	0.24±.04	0.54±.01	0.46±.04
DM10429	*purF gnd hisA1459*	0.44±.02	0.46±.01	0.47±.01
DM11012	*purF gnd hisA10508*	0.46±<.01	0.45±.01	0.51±.01
DM11071	*purF gnd hisA10521*	0.43±.01	0.44±.01	0.50±<.01
DM10973	*purF gnd hisA10505*	0.41±.05	0.44±.01	0.50±.02
DM10975	*purF gnd hisA10506*	0.43±.02	0.44±<.01	0.50±.01
DM10352	*purF gnd hisA1452*	0.43±.01	0.43±.01	0.49±.01
DM11069	*purF gnd hisA1460*	0.44±.01	0.42±.03	0.47±.01
DM10427	*purF gnd hisA1468*	0.43±.03	0.42±.02	0.49±.01
DM10406	*purF gnd hisA1462*	0.43±.01	0.40±.02	0.49±.01
DM10425	*purF gnd hisA1467*	0.41±.01	0.40±.01	0.50±.01
DM10350	*purF gnd hisA1451*	0.34±.01	0.34±.01	0.49±.01
DM11014	*purF gnd hisA10509*	0.35±.03	0.34±.02	0.50±.01
DM10969	*purF gnd hisA10501*	0.26±.01	0.30±.01	0.46±.04

Growth rates (in hours^−1^) are shown and are arranged in descending order by their ability to generate their own histidine (middle data column). All strains were grown in minimal glucose medium at 37°C with adenine and the indicated additions. Thi: thiamine; His: histidine

a NG = no growth; growth rate was <0.03 hours^−1^.

### PRA formation is not caused by de-repressed expression of the histidine operon

Decreasing flux through the biosynthetic pathway can cause histidine limitation which in turn results in de-repressed transcription of the *his* operon [29]. The *hisO1242* mutation [18], which deletes the *his* attenuator and results in a 20-fold increase in transcription of the histidine biosynthetic genes [30], was transduced into a *purF gnd* background. The resulting strain, DM11234 (*purF gnd hisO1242*), did not grow in the absence of exogenous thiamine (i.e., no change in optical density over a 24-hour period at 37°C). This result supported the model that the lesions in *hisA* allowed PurF independent thiamine synthesis by causing altered metabolite pools, not by increasing the flux through the pathway.

### PurF-independent PRA formation is dependent on ProFAR synthesis

Adding exogenous histidine to the medium in the absence of thiamine prevented growth of the *purF gnd hisA* mutant strains (Figure 2). This result suggested flux through the biosynthetic pathway was required for the PurF-independent PRA formation that supported growth of the strains. Null alleles of histidine biosynthetic genes were used to characterize the flux requirements of PurF-independent thiamine synthesis. Histidine reduces metabolic flux by allosteric inhibition of HisG and strains with null mutations in the biosynthetic enzymes require histidine. To reconcile these two facts, a feedback-resistant allele of *hisG* (*hisG1102*) [20] was introduced into the relevant strains to ensure that the only effect on metabolic flux was due to the relevant genetic lesion. A *purF gnd hisG1102* mutant defective in the second step in the pathway (*hisI*) did not grow in the absence of exogenous thiamine (Table 4). Similarly, deletion *hisF109* [31] did not allow a *purF gnd hisG1102* strain to produce sufficient thiamine for growth. In contrast, the *purF gnd hisG1102* strain that carried a null allele of *hisA* (*hisA3000* [31]) grew in the absence of exogenous thiamine. Together these results allowed the conclusion that the formation and accumulation of ProFAR were necessary and sufficient for PurF-independent PRA formation. These data suggested the suppression mechanism of the *hisA* alleles involved facilitating the accumulation of ProFAR while allowing histidine biosynthesis.

**Table 4:** Metabolic flux to ProFAR is required for PurF-independent PRA synthesis.

Strain	Relevant Genotype	+His	+His+Thi
DM728	*purF gnd*	NG^a^	0.48±.01
DM10924	*purF gnd hisG1102*	NG	0.47±.04
DM10923	*purF gnd hisA3000*	NG	0.48±.03
DM10922	*purF gnd hisG1102 hisA3000*	0.34±.05	0.41±.01
DM10928	*purF gnd hisG1102 hisI99*	NG	0.54±.01
DM11136	*purF gnd hisG1102 hisF109*	NG	0.53±<.01
DM10931	*purF gnd hisG1102 his-2652* (Δ*hisCBHAFI*)	NG	0.48±.01
DM10994	*purF gnd hisG1102 his-2652* pSU-*hisI*	0.47±.06	0.51±<.01

Growth rates (in hours^−1^) are shown. Strains were grown in minimal glucose medium with adenine and the indicated additions. His: histidine; Thi: thiamine. Histidine alleles *hisA3000, hisI99, hisF109, his-2652* (*del:CBHAFI*) cause a complete loss of function of the relevant gene product(s). Allele *hisG1102* encodes an enzyme that is insensitive to feedback inhibition by histidine.

a NG = no growth; growth rate was <0.03 hours^−1^.

### ProFAR-dependent PRA formation is not via increased R5P pool size

Growth medium from strain *purF gnd hisA1451* (DM10350) had an ultraviolet (UV) spectrum consistent with the presence of ProFAR (i.e., lambda max at 284 nm) and the supernatant of the *hisA* mutant strain had an increased absorbance at 290 nm compared to the wild-type. Based on the extinction coefficient reported for ProFAR [27], if all of the increase was attributed to ProFAR,

the mutant strain had ∼45 µM more ProFAR in the medium than the isogenic strain *purF gnd* (DM10351) when both were grown in minimal medium with adenine and thiamine. The presence of exogenous ProFAR suggested a parallel endogenous accumulation.

Strain *purF gnd hisA1451* (DM10350) grew in minimal medium with adenine and limiting nitrogen with a doubling time of ∼2 hours. In contrast, the isogenic strain DM10351 (*purF gnd*) failed to grow after 24 hours. A control strain that accumulated R5P and produced PRA by a non-enzymatic synthesis that depended on the ammonia in the medium failed to grow under these conditions without thiamine, as previously reported [1], [4]. These data indicated ProFAR-dependent PRA formation did not simply increase available R5P that reacted with ammonia in the medium. Taken together the above results were consistent with a model in which an increased internal concentration of ProFAR was converted either directly or indirectly into PRA *in vivo*. Thus far, efforts to identify a cellular enzyme that could convert ProFAR to PRA have not been successful. Genetic approaches yielded mutations that affected ProFAR-dependent thiamine synthesis *in vivo* (see below), but failed to identify evidence of an enzyme that converted ProFAR to PRA. Similarly, efforts to detect PRA generated from ProFAR in cell-free extracts were unsuccessful.

### ProFAR breakdown does not generate PRA in vitro

**Figure 3:**
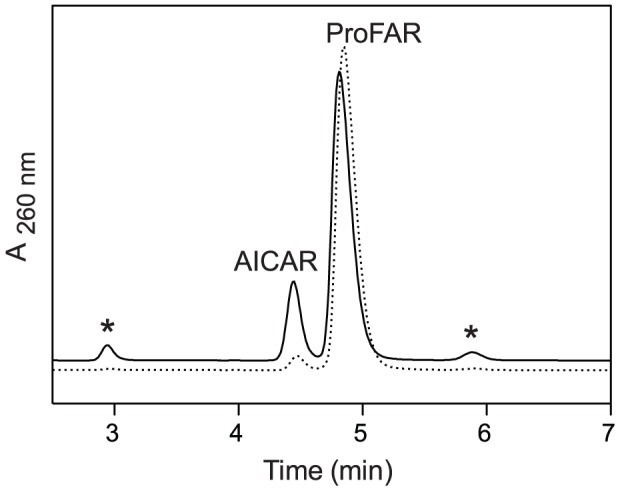
HPLC separation of ProFAR breakdown products. The dashed line indicates the trace of stock 1 mM ProFAR used for this assay. The solid line (offset) indicates the trace of 1 mM ProFAR pH 7.5 after incubation at 37°C for 26 hours. Stars indicate unknown break down products. Abbreviations: AICAR, 5-amino-4-imidazolecarboxamide ribonucleotide; ProFAR, 1-(5-phosphoribosyl)-5-[(5-phosphoribosylamino)methylideneamino]imidazole-4-carboxamide.

*In vitro* at pH 7.5 ProFAR has a half-life of ∼953 min [32]. Davisson *et al*. characterized 5-amino-4-imidazolecarboxamide ribonucleotide (AICAR) as the primary product of ProFAR break down and noted the presence of several other products that degraded further with continued incubation and decreased pH [26]. A solution of ProFAR (1 mM, pH 7.5) was incubated at 37°C for 26 hours, and a 1 mM solution was adjusted to pH 4 and incubated at 45°C for 24 hours. In each case the reaction components were separated by HPLC. The chromatographs of each sample, before and after incubation, were compared (Figure 3 shows the pH 7.5 reactions, before and after incubation). In both samples, after incubation a new peak appeared that was 5-amino-4-imidazolecarboxamide ribonucleotide (AICAR) based on UV spectrum and co-injection with an authentic standard. Two additional minor peaks that appeared in the samples were not identified. After incubation at pH 4, the sample contained less than 3% of the original ProFAR (data not shown).

The presence of PRA in the ProFAR solution was queried by a coupled assay that combine the unstable PRA with glycine via PRA-glycine ligase (PurD) to form stable product glycinamide ribonucleotide (GAR) [24], [33]. After allowing ProFAR degradation at both pH 4 and 7.5 for ∼24 hours, the pHs were adjusted to 8 and the coupled assay performed. Thin-layer chromatography and liquid chromatography mass spectral (LC/MS) analysis failed to detect any GAR. These results supported the conclusion that neither PRA, nor R5P and ammonia were generated by non-enzymatic breakdown of ProFAR.

### Alterations in the metabolic network can impact the ProFAR-dependent PRA formation

Mutations that allowed a *purF gnd hisA1451* strain (DM10350) to grow in the absence of thiamine, despite the presence of 100 µM histidine, were isolated. We anticipated that these mutations would alter flux through histidine biosynthesis and thus impact the accumulation of ProFAR. Consistent with this expectation, the most frequent mutations that allowed growth were in *hisG* and resulted in variants insensitive to allosteric inhibition by histidine. To prevent isolation of these *hisG* mutations, mutant screens were refined to require sensitivity to thiazolealanine, a false feedback inhibitor of HisG [21]. Strains that remained sensitive to thiazolealanine retained an allosterically regulated HisG. Null mutations in two loci were independently isolated several times with this refined screen. Lesions in either *yciB*, which encodes an inner membrane protein essential for intracellular cell division in *Shigella flexneri* [34], [35], or in *proY*, which encodes a cryptic proline transporter [36], allowed growth of the *purF gnd hisA1451* strain in the absence of thiamine when histidine was present. The lesions identified had no obvious connection to histidine flux, and the specific mechanism(s) of the suppression were not pursued further. Nonetheless, the phenotypes identified here will contribute to future studies, both on defining the function of these gene products and extending our understanding the integration of points in the metabolic network.

## Discussion

This study described a new metabolic link between histidine and thiamine biosynthesis in *S. enterica*. Specifically, the data showed that lesions in the histidine biosynthetic gene *hisA* allowed PurF-independent growth in the absence of exogenous thiamine. Based on results of *in vivo* and *in vitro* experiments, we suggest that the conversion of ProFAR to PRA is the mechanism that supports growth of *purF* mutants in the absence of exogenous thiamine. ProFAR is a stable metabolite (half-life was 953 min at pH 7.5) [32], and it is reasonable to suggest that an enzyme would be required to break down ProFAR and generate enough PRA to satisfy the growth requirement for thiamine. Such an enzyme would not need to be highly efficient at generating PRA, since the cellular requirement for thiamine is less than 10 nM [37].

**Figure 4:**
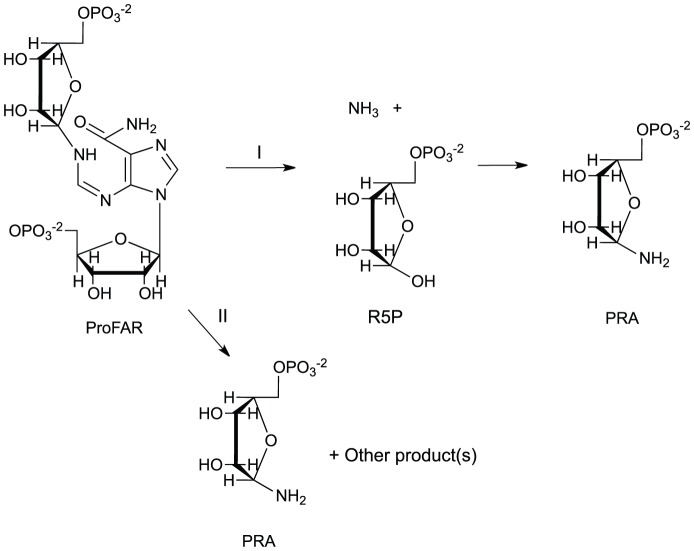
Possible mechanisms for PRA formation from ProFAR. General mechanisms for PRA formation from ProFAR are depicted schematically. In pathway I, ProFAR is hydrolyzed to generate R5P by a mechanism that likely requires an enzyme. Ammonia is also released from the non-R5P product and is then available for non-enzymatic formation of PRA. It is possible the R5P and/or the ammonia do not leave the active site of the relevant enzyme. Pathway II depicts the formation of PRA as a direct product and implicates an undefined enzyme-catalyzed mechanism. Abbreviations: ProFAR, 1-(5-phosphoribosyl)-5-[(5-phosphoribosylamino) methylideneamino] imidazole-4-carboxamide; R5P, ribose-5′-phosphate; PRA, phosphoribosylamine.

In considering the possible chemical mechanisms that would allow PRA formation from ProFAR, we were influenced by the TrpD-mediated mechanism [38]. We propose that the PRPP-derived phosphoribosyl ring of ProFAR could have one of two possible fates: it could be cleaved from the ProFAR with the amino group attached, forming PRA directly, or it could undergo hydrolysis and release R5P. A rearrangement of the rest of the ProFAR moleculecould release free ammonia, which would combine with R5P and non-enzymatically generate PRA (Figure 4). Significantly, the mechanism of PRA formation allowed by ProFAR did not require excess ammonia in the growth medium, suggesting either PRA is the direct product or that both R5P and ammonia are derived from the same molecule during the reaction (Figure 4). The former option is akin to a recently described mechanism whereby TrpD generated PRA directly and did not require R5P and ammonia intermediates [38]. Aside from PurF, TrpD is the only other enzyme that has been shown to generate PRA directly in the cell, but several processes that affect the levels of R5P have been characterized.

As part of this study, several mutants with lesions in genes involved in diverse cellular functions were isolated based on their impact on the histidine/thiamine system. Both *proY* and *yciB* encode proteins that are predicted to be integral membrane proteins [35], [36], yet the phenotype of the null mutants suggest they are impacting the amount of ProFAR available for PRA formation. Further efforts to dissect the mechanism(s) allowing growth of these strains are likely to extend the network connecting the histidine and thiamine biosynthetic pathways. The continued analyses of metabolic integration, such as those described here increase our understanding of cellular biochemistry and gene function, and add to our ability to model and manipulate the complex system that is the metabolic network of a living cell.

## References

[1] Koenigsknecht MJ, Downs DM (2010) Thiamine biosynthesis can be used to dissect metabolic integration. Trends Microbiol 18: 240–247. 20382023 10.1016/j.tim.2010.03.003 PMC2906612

[2] Shuman HA, Silhavy TJ (2003) The art and design of genetic screens: Escherichia coli. Nat Rev Genet 4: 419–431. 12776212 10.1038/nrg1087

[3] Ramos I, Vivas EI, Downs DM (2008) Mutations in the tryptophan operon allow PurF-independent thiamine synthesis by altering flux *in vivo* . J Bact 190: 815–822. 17557816 10.1128/JB.00582-07 PMC2223571

[4] Koenigsknecht MJ, Fenlon LA, Downs DM (2010) Variants of phosphoribosylpyrophosphate synthetase (PrsA) alter cellular pools of ribose 5-phosphate and influence thiamine synthesis in S. enterica. Microbiology 156: 950–959. 19959576 10.1099/mic.0.033050-0 PMC2889433

[5] Maggio-Hall LA, Dorrestein PC, Escalante-Semerena JC, Begley TP (2003) Formation of the dimethylbenzimidazole ligand of coenzyme B(12) under physiological conditions by a facile oxidative cascade. Org Lett 5: 2211–2213. 12816411 10.1021/ol034530m

[6] Browne BA, Ramos AI, Downs DM (2006) PurF-independent phosphoribosyl amine formation in *yjgF* mutants of *Salmonella enterica* utilizes the tryptophan biosynthetic enzyme complex anthranilate synthase-phosphoribosyltransferase. J Bacteriol 188: 6786–6792. 16980480 10.1128/JB.00745-06 PMC1595518

[7] Lambrecht JA, Browne BA, Downs DM (2010) Members of the YjgF/YER057c/UK114 Family of Proteins Inhibit Phosphoribosylamine Synthesis in Vitro. J Biol Chem 285: 34401–34407. 20817725 10.1074/jbc.M110.160515 PMC2966054

[8] Lambrecht JA, Flynn JM, Downs DM (2012) Conserved YjgF protein family deaminates reactive enamine/imine intermediates of pyridoxal 5′-phosphate (PLP)-dependent enzyme reactions. J Biol Chem 287: 3454–3461. 22094463 10.1074/jbc.M111.304477 PMC3270999

[9] Castilho BA, Olfson P, Casadaban MJ (1984) Plasmid insertion mutagenesis and *lac* gene fusion with mini-mu bacteriophage transposons. J Bacteriol 158: 488–495. 6327606 10.1128/jb.158.2.488-495.1984 PMC215454

[10] Way JC, Davis MA, Morisato D, Roberts DE, Kleckner N (1984) New Tn10 derivatives for transposon mutagenesis and for construction of *lacZ* operon fusions by transposition. Gene 32: 369–379. 6099322 10.1016/0378-1119(84)90012-x

[11] Vogel HJ, Bonner DM (1956) Acetylornithase of *Escherichia coli*: partial purification and some properties. Journal of Biological Chemistry 218: 97–106. 13278318

[12] Davis RW, Botstein D, Roth JR, Cold Spring Harbor Laboratory. (1980) Advanced bacterial genetics. Cold Spring Harbor, N.Y.: Cold Spring Harbor Laboratory. x, 254 p.

[13] Balch WE, Fox GE, Magrum LJ, Woese CR, Wolfe RS (1979) Methanogens: reevaluation of a unique biological group. Microbiol Rev 43: 260–296. 390357 10.1128/mr.43.2.260-296.1979 PMC281474

[14] Gutnick D, Clvo JM, Klopotowaski T, Ames BN (1969) Compounds which serve as the sole source of carbon or nitrogen for *Salmonella typhimurium* LT2. Journal of Bacteriology 100: 215–219. 4898986 10.1128/jb.100.1.215-219.1969 PMC315380

[15] Roberts GP (1978) Isolation and characterization of informational suppressors in *Salmonella typhimurium* [PhD Thesis]. Berkeley: University of California.

[16] Schmieger H (1972) Phage P22-mutants with increased or decreased transduction abilities. Mol Gen Genet 119: 75–88. 4564719 10.1007/BF00270447

[17] Downs DM, Petersen L (1994) apbA, a new genetic locus involved in thiamine biosynthesis in Salmonella typhimurium. J Bacteriol 176: 4858–4864. 7519593 10.1128/jb.176.16.4858-4864.1994 PMC196320

[18] Johnston HM, Barnes WM, Chumley FG, Bossi L, Roth JR (1980) Model for regulation of the histidine operon of *Salmonella* . Proceedings of the National Academy of Sciences of the United States of America 77: 508–512. 6987654 10.1073/pnas.77.1.508 PMC348301

[19] Enos-Berlage JL, Downs DM (1996) Involvement of the oxidative pentose phosphate pathway in thiamine biosynthesis in *Salmonella typhimurium* . J Bact 178: 1476–1479. 8631729 10.1128/jb.178.5.1476-1479.1996 PMC177826

[20] Sheppard DE (1964) Mutants of *Salmonella typhimurium* resistant to feedback inhibition by L-histidine. Genetics 50: 611–623. 14221869 10.1093/genetics/50.4.611 PMC1210680

[21] Moyed HS (1961) Interference with the feed-back control of histidine biosynthesis. J Biol Chem 236: 2261–2267. 13773370

[22] Datsenko KA, Wanner BL (2000) One-step inactivation of chromosomal genes in *Escherichia coli* K-12 using PCR products. Proc Natl Acad Sci USA 97: 6640–6645. 10829079 10.1073/pnas.120163297 PMC18686

[23] Bartolomé B, Jubete Y, Martinez E, de la Cruz F (1991) Construction and properties of a family of pACYC184-derived cloning vectors compatible with pBR322 and its derivatives. Gene 102: 75–78. 1840539 10.1016/0378-1119(91)90541-i

[24] Koenigsknecht MJ, Ramos I, Downs DM (2007) Glutamine phosphoribosylpyrophosphate amidotransferase-independent phosphoribosyl amine synthesis from ribose 5-phosphate and glutamine or asparagine. J Biol Chem 282: 28379–28384. 17686772 10.1074/jbc.M704024200

[25] Kitagawa M, Ara T, Arifuzzaman M, Ioka-Nakamichi T, Inamoto E, et al (2005) Complete set of ORF clones of Escherichia coli ASKA library (a complete set of E. coli K-12 ORF archive): unique resources for biological research. DNA Res 12: 291–299. 16769691 10.1093/dnares/dsi012

[26] Davisson V, Deras I, Hamilton S, Moore L (1994) A Plasmid-Based Approach for the Synthesis of a Histidine Biosynthetic Intermediate. Journal of Organic Chemistry 59: 137–143.

[27] Smith DW, Ames BN (1964) Intermediates in the Early Steps of Histidine Biosynthesis. J Biol Chem 239: 1848–1855. 14213364

[28] Lang D, Thoma R, Henn-Sax M, Sterner R, Wilmanns M (2000) Structural evidence for evolution of the beta/alpha barrel scaffold by gene duplication and fusion. Science 289: 1546–1550. 10968789 10.1126/science.289.5484.1546

[29] Brenner M, Ames BN, editors (1971) The histidine operon and its regulation. New York: Academic Press, Inc. 349–387 p.

[30] Ely B, Fankhauser DB, Hartman PE (1974) A fine structure map of the salmonella histidine operator-promoter. Genetics 78: 607–631. 4615040 10.1093/genetics/78.2.607 PMC1213223

[31] Hartman PE, Hartman Z, Stahl RC (1971) Classification and mapping of spontaneous and induced mutations in the histidine operon of Salmonella. Adv Genet 16: 1–34. 4947105 10.1016/s0065-2660(08)60352-1

[32] Henn-Sax M, Thoma R, Schmidt S, Hennig M, Kirschner K, et al (2002) Two (betaalpha)(8)-barrel enzymes of histidine and tryptophan biosynthesis have similar reaction mechanisms and common strategies for protecting their labile substrates. Biochemistry 41: 12032–12042. 12356303 10.1021/bi026092h

[33] Schendel FJ, Cheng YS, Otvos JD, Wehrli S, Stubbe J (1988) Characterization and chemical properties of phosphoribosylamine, an unstable intermediate in the *de novo* purine biosynthetic pathway. Biochemistry 27: 2614–2623. 2454658 10.1021/bi00407a052

[34] Mac Siomoin RA, Nakata N, Murai T, Yoshikawa M, Tsuji H, et al (1996) Identification and characterization of ispA, a Shigella flexneri chromosomal gene essential for normal in vivo cell division and intracellular spreading. Mol Microbiol 19: 599–609. 8830250 10.1046/j.1365-2958.1996.405941.x

[35] Hong M, Gleason Y, Wyckoff EE, Payne SM (1998) Identification of two Shigella flexneri chromosomal loci involved in intercellular spreading. Infect Immun 66: 4700–4710. 9746567 10.1128/iai.66.10.4700-4710.1998 PMC108578

[36] Liao MK, Gort S, Maloy S (1997) A cryptic proline permease in Salmonella typhimurium. Microbiology 143 (Pt 9): 2903–2911. 10.1099/00221287-143-9-2903 9308174

[37] Dougherty MJ, Downs DM (2006) A connection between iron-sulfur cluster metabolism and the biosynthesis of 4-amino-5-hydroxymethyl-2-methylpyrimidine pyrophosphate in Salmonella enterica. Microbiol 152: 2345–2353. 10.1099/mic.0.28926-0 16849799

[38] Lambrecht JA, Downs DM (2012) Anthranilate phosphoribosyl transferase (TrpD) generates phosphoribosylamine for thiamine synthesis from enamins and non-enzymatic chemistry. Chem Biol in review. 10.1021/cb300364k PMC3549051 23101964

